# How long before hospital admission do the symptoms of heart failure decompensation arise?

**DOI:** 10.1590/1518-8345.2735.3119

**Published:** 2019-01-31

**Authors:** Maraísa Carine Born, Karina de Oliveira Azzolin, Emiliane Nogueira de Souza

**Affiliations:** 1 Universidade Federal de Ciências da Saúde de Porto Alegre, Porto Alegre, RS, Brazil.; 2 Universidade Federal do Rio Grande do Sul, Escola de Enfermagem, Porto Alegre, RS, Brazil.

**Keywords:** Heart Failure, Intensive Care Units, Nursing, Nursing Care, Signs and Symptoms, Self Care, Insuficiência Cardíaca, Unidade de Terapia Intensiva, Enfermagem, Cuidados de Enfermagem, Sinais e Sintomas, Autocuidado, Insuficiencia Cardíaca, Unidades de Cuidados Intensivos, Enfermería, Atención de Enfermería, Signos y Síntomas, Autocuidado

## Abstract

**Objective:**

to identify the signs and symptoms of decompensation of heart failure (HF) and the duration of time to hospital admission.

**Method:**

this is a cross-sectional study with adult patients hospitalized for decompensated HF in a teaching hospital located in southern Brazil from July to October 2017. Data collection was performed through a structured questionnaire that included sociodemographic, clinical signs and symptoms of HF. In the data analysis, the following tests were applied: t-Student, Mann Whitney U-Test, Chi-Square Tests.

**Results:**

94 patients, aged between 42 and 98 years old (mean of 71.2 years old) were included. The most prevalent signs and symptoms before emergency admission were dyspnea (79.8%), cough (29.8%), orthopnea (27.7%), edema (23.4%), and fatigue (22.3%). The median time from onset of signs and symptoms to arrival in the emergency room is fatigue and edema 7 days, orthopnea 5 days, cough 4 days and dyspnea 3 days.

**Conclusion:**

the set of classic signs and symptoms of decompensation of HF occurs around seven days before the emergency search and dyspnea is the worsening that leads the patient to a hospital emergency.

## resumo

**Objetivo:**

identificar os sinais e sintomas de descompensação da insuficiência cardíaca (IC) e o tempo de duração até a admissão na emergência hospitalar.

**Método:**

estudo transversal, com pacientes adultos, internados por descompensação da IC em um hospital de ensino localizado no sul do Brasil, no período de julho a outubro de 2017. A coleta de dados foi realizada através de um questionário estruturado que compreendeu dados sociodemográficos, clínicos e sinais e sintomas de IC. Na análise de dados, foram aplicados os testes *t-Student*, *Mann Whitney U Test* e *Chi-Squere Tests*.

**Resultados:**

foram incluídos 94 pacientes, com faixa etária entre 42 e 98 anos (média de 71,2 anos). Os sinais e sintomas mais prevalentes antes da admissão na emergência foram dispneia (79,8%), tosse (29,8%), ortopneia (27,7%), edema (23,4%) e cansaço (22,3%). A mediana de tempo de início dos sinais e sintomas até a chegada na emergência é: cansaço e edema 7 dias, ortopneia 5 dias, tosse 4 dias e dispneia 3 dias.

**Conclusão:**

o conjunto de sinais e sintomas clássicos da descompensação de IC ocorrem em torno de sete dias antes da procura pela emergência e é a piora da dispneia que leva o paciente a uma emergência hospitalar.

## RESUMEN

**Objetivo:**

identificar los signos y síntomas de descompensación de la insuficiencia cardíaca (IC) y el tiempo de duración hasta la admisión en la emergencia hospitalaria.

**Método:**

estudio transversal, con pacientes adultos, internados por descompensación de la IC en un hospital de enseñanza ubicado en el sur de Brasil, en el período de julio a octubre de 2017. La recolección de datos fue realizada a través de un cuestionario estructurado que comprendió datos sociodemográficos, y los signos y síntomas de IC. En el análisis de datos, se aplicaron las pruebas: *t-Student, Mann Whitney U Test, Chi-Square Tests*.

**Resultados:**

fueron incluidos 94 pacientes, con edad entre 42 y 98 años (promedio de 71,2 años). Los signos y síntomas más prevalentes antes de la admisión en la emergencia fueron disnea (79,8%), tos (29,8%), ortopnea (27,7%), edema (23,4%), y cansancio (22,3) %). La mediana de tiempo de inicio de los signos y síntomas hasta la llegada en la emergencia es: cansancio y edema 7 días, ortopnea 5 días, tos 4 días y disnea 3 días.

**Conclusión:**

el conjunto de signos y síntomas clásicos de la descompensación de IC ocurren alrededor de siete días antes de la búsqueda por la emergencia y es el empeoramiento de la disnea que lleva al paciente a una emergencia hospitalaria.

## Introduction

Heart failure (HF) has been a serious and progressive public health problem, leading to an increase in hospital readmissions, a risk of early mortality, a significant impact on the quality of life of patients, and an important source of high costs for the health system, whose elderly population is growing^1^ ^-^ ^2^. It is characterized as one of the main causes of hospitalization in adults, the first cause of hospitalization in elderly patients^3^ ^-^ ^4^, and the main cause of rehospitalization according to the Brazilian Registry of Acute Heart Failure (BREATHE), study that outlined the scenario of HF in Brazil^5^. The reasons occur specifically by three factors: population aging; increased incidence of precursor diseases such as systemic arterial hypertension (SAH), obesity, diabetes mellitus (DM); and improved therapeutics in several cardiac disorders such as acute myocardial infarction (AMI) and valvulopathies, which allows an increase in the survival of these patients, who eventually develop HF over the years^5^.

Although the advances in optimal clinical and therapeutic management have been observed, HF remains a challenge for the health team, resulting in physical and psychological suffering^2^, caused by the lack of knowledge of non-pharmacological measures, inadequate adherence to medication treatment, and by the inability to identify and interpret the signs and symptoms of disease decompensation^4^ ^-^ ^6^.

Patients hospitalized for acutely decompensated HF had until recently a poor prognosis, but better identification of symptoms and therefore more appropriate treatments led to a 40-50% reduction in mortality in a short period of time. Therefore, nursing actions aimed at educating patients and their families for the early recognition of signs and symptoms, indicators of worsening, are important to seek immediate medical attention without the loss of critical time, hours or even before adequate treatment^5^.

It is known that the late recognition of these clinical manifestations is associated with an increase in rates of hospitalizations and complications, mainly including mortality. About two-thirds of these acute events occur in patients with known HF, and about one third is the first event in individuals without the diagnosis of HF^7^ ^-^ ^8^.

Incomplete understanding of the process of identification, interpretation of signs and symptoms of HF, and decision making by patients can be a barrier to the success of professional interventions aimed at effective self-care and prevention of readmission. Although some studies^9^ ^-^ ^11^ have already evaluated patients’ experiences, including how signs and symptoms were interpreted and managed before hospitalization, there is a gap in examining the time between the onset of these clinical manifestations and the search for a service emergency room. This study aims to explore the relationship between the duration of signs and symptoms that patients associate with worsening HF, being in their homes, before seeking health care in a hospital environment. The identification of the mean time of onset of the symptoms that lead the patients to seek an emergency service is a relevant data to compose the set of information that professionals must consider to offer interventions directed to the management of HF.

With the results, it is expected to highlight the relevance to the clinical practice of the nurse, instigating to develop learning interventions to the patients, so they can know how to evaluate, recognize and manage their signs and symptoms of decompensated HF antecedents of the moment of hospitalization, seeking early care. Thus, the objective of this study was to identify the signs and symptoms of decompensation of HF and the duration of the period until hospital admission.

## Method

This is a cross-sectional study with quantitative approach performed with adult patients with HF diagnosis hospitalized for decompensation in a hospital complex located in southern Brazil. Patients aged ≥ 18 years old, of both genders, with medical diagnosis of decompensated HF, were included. Patients with psychiatric disorders and degenerative neurological diseases, patients with hemodynamic instability at admission, and patients in the recent postoperative period (up to three postoperative months) were not included in the study.

A prevalence of 94% of dyspnea was estimated^12^, with a precision and significance of 5%, and the minimum number of participants was 87. In the period from July to October 2017, data collection was performed through a structured questionnaire that comprised sociodemographic, clinical data and signs and symptoms of HF. For sociodemographic characterization, age, gender, race, education level, marital status, employment status, and origin were registered. A health plan, intensive care unit admission (ICU) and in-hospital mortality were found to characterize hospital admission. In the clinical profile, the time of illness and hospitalization in the last year for decompensated HF according to the patient’s own report, presence of comorbidities, etiology and functional class of HF according to New York Heart Association (NYHA) criteria were identified^13^. Also, left ventricular ejection fraction (LVEF) was recorded according to the last echocardiogram, presence, and duration of signs and symptoms of HF experienced before hospital admission according to the patient’s report.

Data collection was performed by the trained researcher who approached the patient to participate in the study. The patients were approached within 48 hours of hospital admission. Those who agreed to participate completed the structured questionnaire in an average time of 15 minutes. After the collection with the patient, some information was checked in the electronic medical records of the hospital, in a reserved place. The data were tabulated in the Excel for Windows worksheet and later exported, processed and analyzed in the Statistical Package for Social Science (SPSS), version 23.0. Descriptive and analytical techniques were adopted for statistical analysis. Continuous variables were expressed as mean and standard deviation or median and percentiles (25-75), and categorical variables, with absolute (n) and relative (%) frequencies. For the normality of the data, the Kolmogorov Smirnov test was applied. The study variables were compared using Student’s t-test and Mann Whitney U Test for independent samples as they followed the normal distribution. The association of sociodemographic and clinical variables was evaluated using the Chi-Square test. The results were considered statistically significant if p <0.05, with 95% confidence interval.

The study was approved by the Research Ethics Committee of the Hospital Irmandade Santa Casa de Misericórdia in Porto Alegre under opinion 2,157,007/2017. The participants received verbal and written information from the research and signed the Informed Consent Term. The ethical precepts of Resolution 466/2012 of the National Health Council^14^ were respected.

## Results

A total of 94 patients, aged between 42 and 98 years old, with a mean age of 71.2±13.4 years old, 53.2% were male, 98.9% were white, and 64.9% had incomplete elementary school. Table 1 shows the sociodemographic characteristics of the sample.

**Table 1:** Sociodemographic characteristics of patients with heart failure. Porto Alegre, RS, Brazil, 2017

**Variable**	**n(%)**
**Age (years old)**	71.2 ±13.4*
**Age group**	
**>60 years old**	**72(76.6)**
**<60 years old**	**22(23.4)**
**Gender**	
**Male**	**50(53.2)**
**Education level**	
**Incomplete Elementary school**	**61(64.9)**
**Complete High School**	**25(26.6)**
**Race**	
**White**	**93(98.9)**
**Marital status**	
**Married**	**56(59.6)**
**Widow**	**27(28.7)**
**Single**	**8(8.5)**
**Separate/divorced**	**3(3.2)**
**Job situation**	
**Retired**	**68(72.3)**
**Active**	**21(22.3)**
**On leave**	**5(5.3)**
**Health insurance**	
**Additional Plan**	**50(53.2)**
**Unified Health System (SUS)**	**44(46.8)**

* Variable described as mean; ± standard deviation

In the last 12 months, 29.4% of 54.3% patients analyzed readmitted for decompensated HF, were hospitalized at least twice. Of the study participants, 42.6% went to the ICU, with in-hospital mortality of 13.8%.

Regarding clinical characteristics, the prevalence of participants in functional classes II and III (45.7% and 46.8%, respectively) was observed. The most frequent HF etiology was ischemia (38%). The mean of the LVEF in the 83 patients with an echocardiogram report was 48.3±16.1, ranging from 20% to 81%. Table 2 shows the clinical characteristics of the sample.

**Table 2:** Clinical characteristics of patients with heart failure. Porto Alegre, RS, Brazil, 2017

**Variable**	**n(%)**
**Disease duration (years)**	18.2±15.1*
**Last year hospitalization for decompensated CI** ^**†**^	
**Yes**	**51(54.3)**
**If so, how many times**	2.1±1.4*
**No**	**43(45.7)**
**Etiology**	
**Ischemic**	**38(40.4)**
**Hypertensive**	**24(25.5)**
**Valvar**	**20(21.3)**
**Idiopathic dilated**	**11(11.7)**
**Chagasic**	**1(1.1)**
**Functional class (NYHA)** ^**‡**^	
**II**	**43(45.7)**
**III**	**44(46.8)**
**IV**	**7(7.4)**
**EF** ^**§**^ **(%)**	48.3±16.1*
**Presence of comorbidities**	
**Arterial hypertension**	**71(75.5)**
**Ischemic Heart Disease**	**50(52.3)**
**Diabetes Mellitus**	**36(38.3)**
**Smoking**	**36(38.3)**
**Chronic Renal Insufficiency**	**23(24.5)**
**Chronic obstructive pulmonary disease**	**23(24.5)**
**Dyslipidemia**	**20(21.3)**
**Valvopathy**	**19(20.2)**
**Alcoholism**	**12(12.8)**
**Depression**	**9(9.6)**
**Cancer Treatment**	**6(6.4)**

* Variables described as mean; ± standard deviation.^†^CI: cardiac insufficiency; ^‡^NYHA: *New York Heart Association*; ^§^EF: ejection fraction: value calculated with a total of 83 patients with an echocardiogram report.

The most prevalent signs and symptoms before hospital admission were dyspnea (79.8%), cough (29.8%), orthopnea (27.7%), edema (23.4%) and fatigue (22.3%). Table 3 shows the relation of signs and symptoms and their duration before hospital admission reported by the patients.

**Table 3:** Presence of signs and symptoms before hospital admission. Porto Alegre, RS, Brazil, 2017

**Variables**	**Presence n(%)**	**Duration in days md(p25-p75)**
**Dyspnea**	**75(79.8)**	**3(1.0-7.0)**
**Cough**	**28(29.8)**	**4(2.0-7.0)**
**Orthopnea**	**26(27.7)**	**5(1.0-14.0)**
**Edema in the legs, ankles, and/or feet**	**22(23.4)**	**7(3.0-14.0)**
**Tiredness**	**21(22.3)**	**7(2.5-15.0)**
**Angina**	**12(12.8)**	**1(1.0-1.75)**
**Weakness**	**8(8.5)**	**1(1.0-6.25)**
**Cognitive problems**	**8(8.5)**	**2(1.25-5.75)**
**Dizziness**	**8(8.5)**	**1(1.0-1.75)**
**Nausea and vomiting**	**7(7.4)**	**2/(1.0-3.0)**
**Pallor and sweat**	**5(5.3)**	**3/(1.5-4.0)**
**Ascites**	**4(4.3)**	**1/(1.0-5.5)**
**Palpitation**	**4(4.3)**	**1/(1.0-1.75)**

The association between the most prevalent signs and symptoms before the admission to the emergency room and the health insurance, before the hospitalization in the last year and the ejection fraction was found to be a significant association between the presence of edema and previous hospitalization (p=0.047). Data is presented in Table 4.

**Table 4:** Association of signs and symptoms more prevalent with health insurance, hospitalization in the last year for decompensated heart failure and ejection fraction. Porto Alegre, RS, Brazil, 2017

**Variable**	**Health insurance** **md(p25-p75)**			**Last year hospitalization for decompensated heart failure md(p25-p75)**		**Ejection Fraction** **md(p25-p75)**
	SUS*	**Additional**	**Hospitalized**	**Not hospitalized**	**EF** ^**†**^ **≤ 45%**	**EF** ^**†**^ **> 45%**
**Dyspnea** ^**‡**^	**3(1.0-7.0)**	**3.5(1.0-8.5)**	**3(1.0-7.0)**	**4(2.0-7.0)**	**3.5(1.0-14.0)**	**4(1.75-7.0)**
**p-value**	**0.605**			**0.385**		**0.979**
**Cough** ^**§**^	**6.5(1.5-7.75)**	**3.5(2.0-7.0)**	**3.5(2.75-8.75)**	**5(1.75-7.0)**	**1.5(1.25-7.0)**	**5.5(3.0-7.0)**
**p-value**	**0.224**			**0.306**		**0.921**
**Orthopnea** ^**‡**^	**3.5(1.0-9.5)**	**7(1.0-60.0)**	**3(1.0-7.0)**	**7.5(2.5-26.0)**	**5(1.0-25.5)**	**7(3.0-19.0)**
**p-value**	**0.347**			**0.123**		**0.734**
**Edema** ^**§**^	**7(4.0-14.0)**	**7(3.0-13.5)**	**6(3.0-11.5)**	**10.5(6.5-18.0)**	**4(2.5-14.0)**	**7(3.0-13.25)**
**p-value**	**0.263**			**0.047**		**0.553**
**Tiredness** ^**‡**^	**9(2.5-45.0)**	**7(2.0-14.0)**	**3(1.5-14.5)**	**14(4.75-30.0)**	**14(2.5-15)**	**4(2.0-30.0)**
**p-value**	**0.605**			**0.161**		**0.705**

*SUS: Unified Health System; ^†^EF: Ejection Fraction; ^‡^Test: *Mann Whitney U Test*; ^§^Teste:*t- Student*.

## Discussion

This study identified the occurrence of signs and symptoms of decompensated HF and the duration of time until the patient sought a hospital emergency service.

Regarding the occurrence of signs and symptoms before the hospital admission, the intensification of dyspnea was the most prevalent. This finding is in agreement with the other studies that proved to be the most important cause of readmission^1^ ^-^ ^3^ ^,^ ^15^ ^-^ ^16^. Other signs and symptoms were also evidenced in this study: cough, orthopnea, edema, and fatigue. Although these signs and symptoms appear with greater intensity before the search for hospital care, orthopnea is more characteristic for HF, since the others can also be found in other clinical conditions^17^. Data from the international literature point out the importance of recognizing HF symptoms, characterized as a complex phenomenon since patients present a variety of symptom combinations at different time intervals, charactered as a mixture of acute and chronic symptoms^18^.

Another study shows that the ability of patients to recognize, interpret and assess worsening symptoms of HF is limited. Suffering and intensification of symptoms such as dyspnoea, edema, and orthopnea are related to the greater delay in care. These delays in seeking care vary from hours to days from onset of symptoms to hospital admission^19^. The identification, tolerance, and decompensation of signs and symptoms caused these patients to seek hospital care within a period of up to seven days. These data are similar at the time of presenting symptoms before hospital admission^12^ ^,^ ^20^ ^-^ ^21^. The mean duration of early symptoms of decompensated HF was one to nine days, but duration of dyspnea ranges from one to eight, 5 days before searching for service.

It is known that symptoms such as edema, weight gain, and fatigue are tolerated by patients with HF around seven days and dyspnea for three days before seeking a health service^9^ ^,^ ^22^. These data is similar to our results because the demand for care was within seven days for the clinical manifestations of worsening HF, and dyspnea in three days. It is inferred that the predominance of these symptoms can vary from 24 hours to two weeks, although most report the presence of the symptom of up to seven days, but there were cases in which the patient took more than 60 days to seek medical attention, the which may have aggravated their clinical condition, with a higher risk of severe complications, including death.

In our study, more than half of the patients hospitalized for decompensated HF were readmitted to the hospital in the last 12 months. This is similar to another study^23^, which showed that 60% are readmitted in 90 days. In the national registry of HF, a study called BREATHE, approximately half of all patients hospitalized with this diagnosis are readmitted within the first 90 days after hospital discharge, and this hospital readmission is considered a risk factor for death^5^.

Together with the high rates of readmissions, the high in-hospital mortality rate was 13.8% of patients admitted for HF decompensated in this study, higher than the mortality rate in the BREATHE registry (12.6%)^5^, and in the DATASUS records, which is 11.0%^24^. These data are more than twice that found in US and European registries: 4.0% in-hospital mortality in the ADHERE study and 3.8% in the Euro Heart Survey^5^.

It is observed that most of the causes of hospitalization are associated with multiple other aggravating risk factors^5^. The presence of multiple comorbidities associated with HF, as shown in the results of this study, may contribute to complications and accelerate decompensation, associated or not with preserved EF (>45%). Consequently, a longer hospital stay may be required to compensate for HF, requiring ICU admission, and a worse prognosis for in-hospital death. Despite efforts by researchers to identify factors associated with frequent hospital readmissions, it is not possible to identify the real reason for clinical decompensation in 30% to 40% of cases^5^.

In view of these findings, it is possible to infer that patients are slow to recognize or even know the exacerbation and worsening of HF to seek hospital care, since the mortality rate and the worsening were high, with a relatively high percentage of ICU admission.

Therefore, the fact that HF is a chronic and progressive disease should be highlighted, that the patient should be involved in the identification of the signs and symptoms of HF, thus being able to communicate the family/companion and seek hospital care earlier, as delays in seeking medical care worsen the clinical situation.

Therefore, it is necessary to better understand how patients identify and value the occurrence of clinical manifestations of worsening of the disease, considering the presence of comorbidities, such as the identification of signs and symptoms that lead them to seek health care in the environment to be able to draw up strategies that aim to achieve and maintain the clinical stability of these patients through a personalized education for self-care. Effective self-care has been recognized as an important and indispensable component in the recognition of CI decompensation^8^.

Measures can be instituted in these readmissions, taking advantage of the impact caused by hospitalization and the symptoms of decompensation. Early hospital discharge planning can be performed, including daily visits to assess and reinforce adherence, support patient and family members, and emphasize the early recognition of signs and symptoms of worsening. This approach can be used successfully to decrease or prevent further hospital admissions. It should be emphasized that the data of this study point to the need to highlight, together with patients and family members, the most characteristic signs and symptoms of HF decompensation. According to the results, it is believed that in the presence of at least one of the signs and symptoms that precede resting dyspnea, such as tiredness, edema, orthopnea, and cough, the patient already needs a professional support, which is not necessarily a hospital emergency, but rather in primary care services or CI management programs.

In this context, the nurse performs an important role in planning actions for the social and health challenges related to the aging of the population and the significant increase in the prevalence of non-communicable chronic diseases. Continuous follow-up of patients with HF is a recommendation of Class 1^8^ in the recent guidelines on the clinical management of HF^25^, and patient engagement is one of the main strategies of the care plan. Thus, nursing interventions to meet the patient’s needs require careful and strategic planning (including an accurate assessment of the needs and support network available), choice of the best approach and an evaluation of the effectiveness of the practice of self-care and its translation into favorable results.

Currently, it is recommended that patients with HF receive multidisciplinary care during their follow-up at different levels of health care. This strategy has greatly reduced hospitalization rates and re-hospitalization due to decompensation and it has had an impact on the quality of life of these patients^8^. The purpose of multidisciplinary follow-up is to teach, reinforce, improve and constantly evaluate self-care skills, which include weight monitoring, hydrosaline restriction, physical activity, drug compliance, sign, and symptom monitoring and the demand for an early health care.

This study brings contributions referring to the main triggers for the search of a hospital emergency service, elucidating the time that the patients remain with the signs and symptoms of decompensation of the CI at home until the search of care. It is important to mention that decision-making in seeking a health service is related not only to the identification and interpretation of signs and symptoms of worsening HF but also to external factors such as access to health services and professionals, previous experiences in terms of problem-solving in settings other than a hospital emergency, and the support resources patients have to travel to a health facility. The best management of HF is through an adequate understanding of the context of the patient to customize the educational interventions so the patient and the family feel engaged in the therapeutic plan.

As a limitation of this study, there were the behavior of variables not evaluated over time to infer causal relationships between them.

## Conclusion

A set of signs and symptoms of HF decompensation, consisting of dyspnea, cough, orthopnea, edema, and fatigue in general, has been present for at least seven days before the search for health care. Among them, dyspnea is the most prevalent and decisive symptom for the patient to seek a hospital emergency service, taking approximately three days.

In this sense, the results can subsidize personalized educational interventions with the aim of helping patients and family members better manage the onset episodes of clinical manifestations of HF decompensation.
